# Recent Developments in β-Cell Differentiation of Pluripotent Stem Cells Induced by Small and Large Molecules

**DOI:** 10.3390/ijms151223418

**Published:** 2014-12-17

**Authors:** S. Suresh Kumar, Abdullah A. Alarfaj, Murugan A. Munusamy, A. J. A. Ranjith Singh, I-Chia Peng, Sivan Padma Priya, Rukman Awang Hamat, Akon Higuchi

**Affiliations:** 1 Department of Medical Microbiology and Parasitology, Universities Putra Malaysia, Serdang 43400, Selangor, Malaysia; E-Mail: rukman@upm.edu.my; 2 Department of Botany and Microbiology, College of Science, King Saud University, Riyadh 11451, Saudi Arabia; E-Mails: aalarfajj@ksu.edu.sa (A.A.A.); ammurugan11@gmail.com (M.A.M.); 3 Department of Bioscience, Jacintha Peter College of Arts and Sciences, Ayakudi, Tenkasi, Tamilnadu 627852, India; E-Mail: ranjitspkc@gmail.com; 4 Department of Chemical and Materials Engineering, National Central University, No. 300, Jhongda RD., Jhongli, Taoyuan 32001, Taiwan; E-Mail: chia80403@hotmail.com; 5 Department of Basic Science and Department of Surgical Sciences, Ajman University of Science and Technology-Fujairah Campus, P.O. Box 9520, Al Fujairah, United Arab Emirates; E-Mail: priyaganu28@gmail.com

**Keywords:** beta (β) cells, diabetes, differentiation, stem cells, pancreas

## Abstract

Human pluripotent stem cells, including human embryonic stem cells (hESCs) and human induced pluripotent stem cells (hiPSCs), hold promise as novel therapeutic tools for diabetes treatment because of their self-renewal capacity and ability to differentiate into beta (β)-cells. Small and large molecules play important roles in each stage of β-cell differentiation from both hESCs and hiPSCs. The small and large molecules that are described in this review have significantly advanced efforts to cure diabetic disease. Lately, effective protocols have been implemented to induce hESCs and human mesenchymal stem cells (hMSCs) to differentiate into functional β-cells. Several small molecules, proteins, and growth factors promote pancreatic differentiation from hESCs and hMSCs. These small molecules (e.g., cyclopamine, wortmannin, retinoic acid, and sodium butyrate) and large molecules (e.g. activin A, betacellulin, bone morphogentic protein (BMP4), epidermal growth factor (EGF), fibroblast growth factor (FGF), keratinocyte growth factor (KGF), hepatocyte growth factor (HGF), noggin, transforming growth factor (TGF-α), and WNT3A) are thought to contribute from the initial stages of definitive endoderm formation to the final stages of maturation of functional endocrine cells. We discuss the importance of such small and large molecules in uniquely optimized protocols of β-cell differentiation from stem cells. A global understanding of various small and large molecules and their functions will help to establish an efficient protocol for β-cell differentiation.

## 1. Introduction

Diabetes mellitus is the most common metabolic disorder with increasing incidence worldwide, predicted to exceed 350 million by 2030 [1]. Currently, conventional therapies are not widely successful because inactive β-cells in the pancreatic islet lead to several associated ailments and system disorders [2,3,4]. In future, stem cell therapy is expected to be more powerful than existing treatments for this pervasive and debilitating disease. Naturally, much attention has been directed to the generation of pancreatic β-cells without tumor formation or immune rejection from human embryonic stem cells (hESCs) in the last few years.

A new generation of research has recently focused on pluripotent stem cells similar to ESCs, known as induced pluripotent stem cells (iPSCs), which are derived from adult somatic cells by inducing expression of certain pluripotency (stem cell) genes, such as *OCT3/4*, *SOX*2, *c-MYC*, and *KLF-4*, or certain miRNAs or proteins (piPS) [5,6,7,8,9]. The human iPSCs (hiPSCs) can be derived from various non-pluripotent cells, such as adipose cells, amniotic fluid cells, hepatocytes, blood cells, fibroblasts, and bone marrow cells [9,10,11,12,13,14]. These hiPSCs have self-renewal and gene expression characteristics similar to those of ESCs and are less problematic in terms of ethical issues [15].

Several studies so far have been published on the generation of pancreatic cells from hESCs [16,17,18,19,20,21,22,23]. However, they do not describe systematic methodologies to differentiate human pluripotent stem cells (hESCs and hiPSCs) and human mesenchymal stem cells (hMSCs) into β-cells. In this review, we will focus on potential problems with various methodologies and discuss how small and large molecules promote the differentiation of hESCs, hiPSCs, and hMSCs into β-cells. It is also important to touch on islet cell therapy, which has its own advantages over pancreatic transplantation in fighting type I diabetes. However, islet cell therapy is not our focus in this review. We will also discuss the signaling pathways involved in β-cell differentiation.

## 2. Importance of β-Cell Differentiation

**Figure 1:**
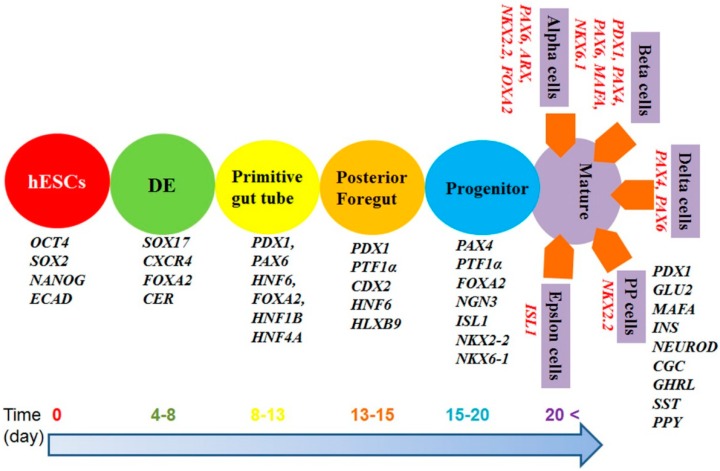
Timeline of differentiation of pluripotent stem cells into β-cells and expression of several genes.

There are three different stages during pancreatic cell differentiation: specification, expansion, and differentiation. hESCs differentiate into insulin-producing cells through stages in the following order: Definitive endoderm (DE) pancreas specification such as primitive gut tube and pancreatic foregut, pancreas progenitor development, and development of mature differentiated β-cells characterized by expression of various transcription factors (Figure 1 and Table 1). The human pancreatic system contains one million active islets; Each islet has approximately 6–8 × 10^6^ β-cells [24]. Failure or absence of insulin-producing cells as a result of the auto-immune destruction of β-cells in these islets leads to type I diabetes mellitus. Currently, insulin injection treatment and pancreatic islet cell transplantation are the only effective treatments for type I diabetes. However, pancreatic islet cell transplantation is not widely successful because of immune rejection and the shortage of donors [25,26]. Another practical disadvantage of transplantation is that 2 × 10^6^ of β-cells per kg of patient body weight are necessary to achieve good metabolic control by production of insulin in type I diabetes [27]. Moreover, there has been no controlled clinical trial to determine the amount of human fetal pancreas and the number of transplantations needed to achieve insulin production in type I diabetes [26,28]. Thus, regenerative medicine provides increased potential to treat diabetes mellitus. Soria *et al.*, implanted approximately 1 × 10^6^ clusters of insulin-producing cells differentiated from hESCs into the spleens of diabetic mice and found that this treatment resolved hyperglycemia and restored normal body weight within a week [29]. Similarly, Jiang *et al*., transplanted 1 × 10^6^ β-cells differentiated from hESCs into the left renal capsule of diabetic mice and observed that 30% of mice showed euglycemia, and the remaining 70% showed hyperglycemia [30]. Rezania *et al.*, also injected 1.9 × 10^6^ of β-cells differentiated from hESCs into mice and found that the α-cell mass significantly decreased, but secretion of glucagon and insulin responded to physiological stimuli [31].

**Table 1:** Overview of different transcription factors expressed during various stages of β-cells differentiated from pluripotent stem cells. “** [ ]” indicates Mesoendoderm. This shows that closed box [ ] contain transcription factor for Mesoendoderm, the rest is definitive endoderm.

References	Initial stage	DE Induction	Pancreas Induction		Differentiation	
		** Mesoendoderm/Definitive Endoderm	Primitive Gut Tube	Posterior for Gut	Pancreatic Endoderm	Hormone Expressing
		STAGE 1	STAGE 2	STAGE 3	STAGE 4	STAGE 5
[32]	*OCT4*, *NANOG*, *SOX2*, *ECAD*	** [*BRA*, *FGF4*, *WNT3*, *NCAD*] *SOX17*, *CER*, *FOXA2*, *CXCR4 (DE)*	*HNF1β*, *HNF4α*	*PDX1*, *HNF6*, *HLXB9*	*NKX6-1*, *NGN3*, *PAX4*, *NKX2-2*	*INS*, *CGL*, *GHRL*, *SST*, *PPY*
[33] (No Serum)	–	*SOX17*, *FOXA2*, *HNF4α*, *GATA4*, *CXCR4*	*PDX1*, *FOXA2*, *SOX17*, *CXCR4*, *HLXB9*, *PTF1α*, *NGN3*, *NKX6.1*	*PDX1*, *PTF1α*, *NGN3*, *ISL1*, *NKX6-1*	*PDX1*, *CK-19*, *INS*, *Glucagon*, *GLU2*, *ISL1*, *NKX6-1*	*–*
[34]	*FOXA2*, *SOX17*	*PDX1*, *PTF1α*, *NGN3*, *INS*, *Somatostatin*, *Glucagon*, *Amylase*	*PAX4*, *NKX2.2*, *NKX6.1*, *ISL1*, *INS*, *Somatostatin*, *Glucagon*, *Amylase*	*PAX4*, *NKX2.2*, *NKX6.1*, *ISL1*, *INS*, *Somatostatin*, *Glucagon*, *Amylase*	*PAX4*, *NKX2.2*, *NKX6.1*, *ISL1*, *INS*, *Somatostatin*, *Glucagon*, *Amylase*	*–*
[35]	*–*	*BRACHURRY*, *SOX17*, *FOXA2*, *HNF4α*	*HNF4α*	*HNF4α*, *PDX1*	*NGN3*, *PDX1*	*INS*, *C-peptide and glucagon*
[36]	*OCT4*, *NANOG*, *SOX2*, *ECAD*	** [ *BRA*, *FGF4*, *WNT3*, *NCAD (1–2 days)*] *SOX17*, *CER*, *FOXA2*, *CXCR4*	*HNF1B*, *HNF4A*	*PDX1*, *HNF6*, *PROX1*, *SOX9*	*NKX6-1*, *NGN3*, *PTF1A*, *NKX2-2*	*–*
[37]	*–*	*CXCR4*, *SOX17*, *FOXA2*	*PDX1*	*–*	*PDX1*	*-*
[38]	–	*FOXA2*, *CXCR4*, *SOX 17*	*PDX1*, *HNF6*, *PAX6*	*PDX1*, *FOXA2*, *SOX9*, *HNF1B*, *MAFA*, *INS*, *GLU2*, *NKX6-1*, *GLUCOKINASE*, *TCF1*	*–*	*PDX1*, *NKX6-1*, *GLUT2*, *MAFA*, *ISL-1*, *NEUROD*
[39]	–	*SOX17*, *GSC*, *FOXA2*, *CXCR4*	*HNF1β*, *HNF6*	*HNF1b*, *HNF6*, *SOX9*, *HLXB9*, *PDX1*	*NKX6.1*, *NGN3*, *PAX4*, *PDX1*, *FOXA2*	*PDX1*
[40]	*OCT4*	*FOXA2*, *SOX 17*	*HNF1β*, *HNF4α*	*PDX1*	*AMY*	*–*
[41]	–	*CDX2*, *SOX2*, *SOX9*	*–*	*–*	*NGN3*, *ISL1*, *NEUROD1*, *PAX6*, *MAFB*, *PROX1*	*INS*, *GCG*, *SST*, *ARX1*, *MAF*, *INSM1*
[42]	–	*SOX17*, *FOXA2*	*–*	*PDX1*, *HNF6*, *HLXB*, *NGN3*, *NEUROD1*, *SOX9*	*INS*, *C-Peptide, PDX1*, *NEUROD1*, *ISLET-1*, *PAX6*, *and NKX2.2*, *glucagon ghrelin*, *or somatostatin*	*–*
[43]	–	*SOX17*, *GSC*, *FOXA2*, *CXCR4*	*FOXA1*, *HNF1β*, *HNF4α*	*PDX1*, *HNF6*, *PROX1*, *SOX9*	*NKX6-1*, *PTF1α*, *NGN3*, *NKX2-2*	*CHGA*, *INS*, *GCG*, *SST*
[44]	–	*SOX17*, *GSC*, *FOXA2*, *CXCR4*	*FOXA1*, *HNF1β*, *HNF4α*	*PDX1*, *HNF6*, *PROX1*, *SOX9*	*NKX6-1*, *PTF1α*, *NGN3*, *NKX2-2*	*CHGA*, *INS*, *GCG*, *SST*

hMSCs were also isolated from the Wharton’s jelly of the umbilical cord and differentiated into insulin-producing cells, and 2 × 10^6^ cells were transplanted without causing any hypoglycemia [45]. Alipio *et al.*, observed that mouse iPSCs were not only able to develop endogenous insulin-secreting cells but also responded to glucose physiological stimuli; When 2 × 10^6^ differentiated β-cells were injected into mice, they also corrected a hyperglycemic phenotype [46]. Similarly, 5 × 10^6^ differentiated mouse induced pluripotent stem cells iPSCs were implanted into the left subcapsular renal space of nonobese diabetic/severe combined (NOD/SCID) mice, and the blood glucose level of the mice was normalized within four days after transplantation [47]. The quality of hESCs and hiPSCs can be characterized during differentiation *in vitro* without any risk of tumor generation prior to transplantation. Although there is a question of functional β cells derived *in vitro*, the number of real beta cells should be identified from the mixture of beta cells used generally for this scope. A differentiated β-cell culture in a 25-mm petri plate will be large enough for this quality evaluation of differentiated cells as a first screening prior to *in vivo* β-cell studies.

## 3. Signal Transduction Pathways

**Figure 2:**
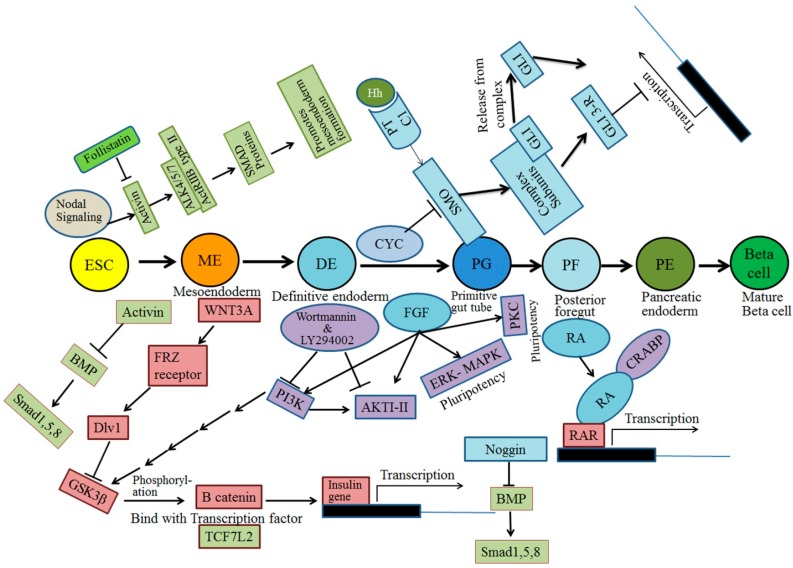
Signaling pathways involved during the differentiation of β-cells from pluripotent stem cells.

The signal transduction pathways involved in pancreatic β-cell differentiation from hESCs have been extensively studied over the last two decades. This section explains the different pathways, along with the respective receptor information, involved in β-cell differentiation, such as Notch signaling, Transforming growth factor signaling, Fibroblast growth factor signaling, WNT signaling, bone morphogenetic protein (BMP) signaling, and retinoic acid receptor signaling (Figure 2). A comprehensive understanding of pancreatic development must distinguish extracellular signals at each stage and also recognize the fundamental molecular mechanisms of each molecule and factors that activate its respective signal to trigger ESCs to differentiate into β-cells. β-cell development also relies on other extracellular signals [48]. Attention has largely focused on the identification of fundamental networks of molecules and signaling pathways in the development of insulin-producing cells.

Several molecules act as extracellular signals for the proper development of the pancreatic cell lineage, in which the first stage of definitive endoderm receives signals from adjacent tissues. At the start of pancreatic development, signals from the TGFβ superfamily of activins play a prime role. Massague and Chen [49] and Frandsen *et al.* [50], indicated that distinct activin subunits form dimers. The presence of activin and the fact that nodal signaling is high at this stage are suppressed by the negative action of the PI3K signaling pathway to activate the pluripotency of hESCs (Figure 2) [51]. Activated PI3K utilizes phosphatidylinositol mono-, di-, or tri-phosphate to activate protein kinase B (PKB otherwise known as AKT) and glycogen synthase kinase. Wortmannin [52,53] and Ly294002 [54] inhibit PI3K [52] and AKTI-II [55] to enhance the differentiation of hESCs into DE. Similarly, PI3K signaling is low and nodal signaling is high to specify DE formation by the activation of activin (Figure 2) [49,56]. Activin A has been demonstrated to play a pivotal role in the migration of pancreatic islets and regulates the differentiation of endocrine and exocrine cells during the initial formation of the pancreas [57,58,59,60,61,62,63].

Great attention has been given to β-cell formation using various small and large molecules, but the extra signaling pathways are not yet clearly understood.

The WNT pathway is another important signaling pathway in pancreatic development, mainly in cell polarity, migration, and proliferation. Whether the WNT pathway promotes self-renewal or differentiation during hESC differentiation and organogenesis is controversial. Approximately 20 different WNT molecules have been identified, among with a few that bind and signal through the Frizzled receptor (FRZ) and activate a protein called DVL to block GSK3β, which phosphorylates β-catenin (Figure 2) [37]. Therefore, unphosphorylated β-catenin accumulated in the cytoplasm forms a complex with transcription factor TCF7L2 at the nucleus (Figure 2) [37]. This complex of β-catenin and transcription factor TCF7L2 is important for the development of the pancreas and its function to secrete insulin. WNT signaling is more important during the initial stage than at the later stages of hESC differentiation. Davidson *et al.*, recently found that *OCT4* repressed WNT pathway signaling during the self-renewal process. β-Catenin signaling was only observed when *OCT4* was knocked out [64]. It was therefore concluded that the WNT signaling pathway mainly functions in the differentiation, but not the self-renewal, of hESCs. Cai *et al.*, also observed that WNT3A could stimulate cell proliferation of hESCs [65]. The accumulation of β-cell signaling in the nuclei occurs and Wnt signaling is not required for hESC pluripotency [36,66].

Several studies suggested that the combination of WNT3A and activin A promotes differentiation of hESCs into definitive endoderm [32,39,67,68]. However, Sato *et al.*, demonstrated that WNT signaling is important for the self-renewal process in both mouse and human ESCs [69]. WNT signaling is therefore likely obligatory in promoting pluripotency during the reprogramming of hiPSCs [33,38].

The BMP signaling pathway also acts as an inhibitor at early stages of endoderm development, whereas it is required in the latter part of pancreatic progenitor formation [55,65,70]. The BMP signaling pathway is controlled by the noggin molecule (Figure 2) [39,71].

Inhibition of the sonic hedgehog pathway in human and mouse cells promotes the formation of the pancreas [72,73,74,75]. In early stages, during the formation of mesoendoderm and definitive endoderm, activin A and FGF2 are used to inhibit the sonic hedgehog pathway [32,71]. Similarly, FGF2 and cyclopamine are used at the progenitor stage [32,76]. The target gene of the sonic hedgehog pathway is expressed during β-cell differentiation from hESCs when the signal is initiated at the Patched (Ptc1) receptor and further triggered by Gli protein through the smoothened (Smo) protein (Figure 2) [77].

## 4. Timelines for β-Cell Differentiation

Researchers are also actively developing efficient protocols and methods for pancreatic differentiation from hESCs, hiPSCs, and hMSCs. The protocols and methods rely on the function of multiple genes and various factors, chiefly small and large molecules that are involved in pancreatic cell differentiation in the human system; Nonetheless, it is difficult to recapitulate pancreatic development *in vitro* [42,78].

**Figure 3:**
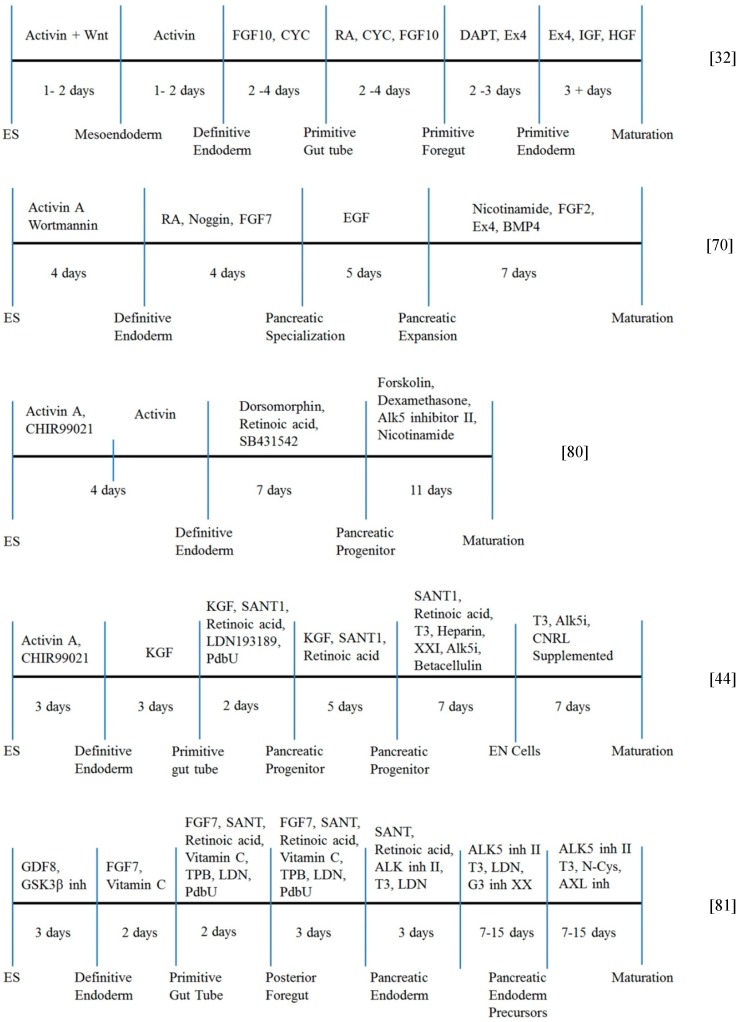
Typical schematic representation of three different timelines during pluripotent stem cell differentiation into β-cells.

Recently, a five-stage protocol was reported comprising the different stages of (a) induction of an initial stage of definitive endoderm; (b) primitive tube formation; (c) development of posterior foregut; (d) development of progenitor cells; and (e) successful production of pancreatic β-cells from human hESCs and hiPSCs *in vitro* (Figure 3). Shi *et al.* [79], Cai *et al.* [65], and Kunisada *et al.* [80], reported that conversion of hiPSCs from fibroblasts to pancreatic β-cells was accomplished through a three-stage differentiation process (Figure 3). In their studies, embryoid bodies were generated from a single-cell suspension of hiPSCs and allowed to undergo further pancreatic differentiation.

Recently Rezania and Kieffer *et al.*, developed a seven-stage protocol, which efficiently generates functional β-cells from hESCs (Figure 3) [81]. Their functional β-cells expressed *MAFA*, *PDX1*, *NKX6.1*, and *NEUROD1*, key markers of mature pancreatic β-cells, and showed glucose-stimulated insulin secretion, which is similar to β-cells in human islets during static incubations *in vitro*.

Introduction of vitamin C at early stages of differentiation successively produced PDX1^+^/NKX6.1^+^ pancreatic progenitors with low expression of *NGN3* and its downstream targets (Stage 4) [81]. Further differentiation of pancreatic progenitors were performed by using a combination of several drugs such as an ALK5 (TGFβ receptor) inhibitor (ALK5 inh II), BMP receptor inhibitor (FGF7, TPB (((2*S*,5*S*)-(*E*,*E*)-8-(5-(4-(trifluoromethyl)phenyl)-2,4-pentadienoylamino)benzolactam), and LDN), and thyroid hormone (T3), which resulted in upregulation of *NGN3* and cell populations co-expressing *PDX1*, *NKX6.1*, *NEUROD1*, and *NKX2.2* (Stage 5) [81]. Continuous exposure of ALK5 inhibitor, BMP receptor inhibitor, and T3 with addition of a notch inhibitor (GSiXX, Gamma secretase inhibitor XX) resulted in the generation of cell populations in which PDX1^+^/NKX6.1^+^/NEUROD1^+^ cells expressed insulin but not glucagon or somatostatin (Stage 6) [81]. Finally, the cells were treated with R428, an inhibitor of AXL in combination with ALK5 inhibitor and T3 induced functional β-cells (MAFA^+^/PDX1^+^/NKX6.1^+^/NEUROD1^+^ cells), which are insulin^+^/glucagon^−^/somatostatin^−^ cells (Stage 7) [81].

The characterization of dynamic glucose stimulation assays revealed similarities and also differences between their functional β-cells and primary human β-cells. Especially, their functional β-cells rapidly returned to diabetes in mice within 40 days, was approximately four times faster than the pancreatic progenitors [81]. Currently, their functional β cells are not fully equivalent to mature human β-cells. However, the capacity for glucose-responsive insulin secretion of their functional β-cells *in vivo* makes them a potential alternative source of pancreatic progenitor cells or cadaveric islets for the clinical treatment of diabetes.

Pagliuca and Melton *et al.*, also developed a six-stage protocol to generate glucose-responsive, monohormonal insulin-producing cells from hESCs and hiPSCs, which expressed coexpression of key β-cell markers and β-cell ultrastructure by using sequential modulation of multiple signaling pathways in a suspension cell culture in a spinner bioreactor (Figure 3) [44]. These stem cell-derived β-cells mimic the function of human islets both *in vitro* and *in vivo*, and secreted human insulin into the blood of mice shortly after transplantation in a glucose-regulated manner for at least 112 days. The transplantation of these stem cell-derived β-cells improved hyperglycemia in diabetic mice, which demonstrated the potential utility of these stem-cell-derived β-cells for *in vivo* transplantation therapy for diabetes [44].

hMSCs obtained from bone marrow or adipose tissues are less expandable than hESC or hiPSC populations (Table 2), but they are also derived from the recipient, similar to hiPSCs, which may lessen the need for immunosuppression in patients. The pancreatic differentiation from hMSCs might also be promising in clinical application.

**Table 2:** Advantages and disadvantages of hMSCs, hESCs, and hiPSCs for differentiation into β-cells.

Advantage and Disadvantage of Clinical Conditions	hMSCs	hESCs	hiPSCs
Ethical concern	no	yes	no
Xeno-free, feeder free culture	easy	difficult	difficult
Preparation of pluripotent (multipotent) stem cells	easy	relatively difficult	relatively difficult
Long-term expansion	difficult	easy	easy
Differentiation ability into β-cells	low	high	high
Tumor generation possibility	no	yes	yes
Mass production for clinical usage	no	yes	yes

## 5. Small and Large Molecules

Pancreatic β-cells develop from the initial formation of ectoderm, mesoderm, and endoderm. Embryonic cells from the endoderm form three different types of gut, classified as foregut, hindgut, and midgut, *in vivo*. The pancreas and other organs such as the liver, gallbladder, and lungs develop from the foregut. The systematic development of the pancreas in humans comprises multiple processes *in vivo* [82].

The three different stages during the differentiation of pancreatic cells from stem cells are specification, expansion, and differentiation. Small and large molecules potentially play an important role in the formation of definitive endoderm and further differentiation into pancreatic tissue (Table 3). Clinical application of gene therapy has been studied using hESCs and hiPSCs induced by small and large molecules for the development of liver or pancreatic islets.

Here, we will discuss specific small and large molecules inducing the differentiation of human stem cells into β-cells in the following sections.

**Table 3:** Overview of small and large molecules involved during various stages of β-cells’ differentiation from pluripotent stem cells. “** [ ]” indicates Mesoendoderm. This shows that closed box “[ ]” contain molecules for Mesoendoderm, the rest is for definitive endoderm.

References	DE Induction	Pancreas Induction		Differentiation			
	** Mesoendoderm/Definitive Endoderm	Primitive Gut Tube	Posterior for Gut	Pancreatic Endoderm	Hormone Expressing		
	STAGE 1	STAGE 2	STAGE 3	STAGE 4	STAGE 5		
[32]	** [Activin A + WNT3A (RPMI) (1–2 days)] Activin A (RPMI) (1–2 days)	FGF10 + CYC (RPMI + FBS) (2–4 days)	RA + CYC + FGF10 (DMEM/B27) (2–4 days)	+/− DAPT + EX4 (DMEM/B27) (2–3 days)	+/− EX4 + IGF1 + HGF (CMRL/B27) (3+ days)		
[71]	Activin A + Sodium butyrate (1 day)	–	EGF + FGF-2 + Noggin (7–14 days) (RPMI/B27)	EGF + Noggin (7 days) (RPMI/B27)	RPMI/bovine serum albumin) (Nicotinamide + IGF-II) (5 days) & without IGF-II for 2 days		
(No Serum)	(RPMI/B27)						
[83]	Activin A + BMP4 (10 days)	FGF18 + B27 (DMEM-F12/B27) (7 days)	FGF18 + B27, (EGF + TGFα + IGFI + IGFII + VEGF) DMEM F12/B27) (7 days)	Forskolin + FBS (HGF + PYY) (10 days)	–		
[84]	Activin A + WNT3A (RPMI) (1 day)	FGF10 + sKAAD-Cyclopamine	All-trans retinoic acid	Betacellulin + Nicotinamide	Betacellulin + Nicotinamide		
	Activin A + FBS (RPMI) (2 days)	(RPMI) (3 days)	FGF10, KAAD-cyclopamine (DMEM/B27)	(DMEM/B27, Gamma SIX + EX-4) (2 days)	(CMRL/B27) (6 days)		
[39]	** [Activin A, WNT3A (RPMI) (1 day)]	KGF (RPMI + FBS) (3 days)	RA, CYC, NOG (DMEM/B27) (3 days)	No factors (DMEM/B27) (3 days)	–		
	Activin A (RPMI, FBS) (2 days)						
[65]	Activin A; Activin A + ITS (2 days)	DE cells were dissociated and replated on mitomycin treated 3T3 Cells in Matrigel plate FGF7 + RA (6 days) (DF12/B27)	(DF12/B27) (KGF + BMP2 + RA + Noggin) (2 days)	Basal medium without KGF, HGF, EX4, Nicotinamide) (6 days)	–		
[70]	Activin A, Wortmannin (DF12) (4 days)	RA + NOGGIN + FGF7 (DF12/IMDM) (4 days)	EGF (5 days)	Nico + FGF-2 + EX4 + BMP4 (DF12) (7 days)	–		
[68]	** [Activin A, WNT3A]; Activin A + FBS (RPMI)	FGF10, KAAD, Cyclopamine (3 days)	FGF10, KAAD, Cyclopamine, RA, Noggin	NA, EX4, IGF1, HGF	–		
			EX4 + Gamma secretase inhibitor compound E (4 days)				
[85]	Activin A, BMP4, FGF-2; Matrigel (3–4 days)	–	FGF-2 + ITS (14 days)	Serum free-ITS, FINE, FGF7, Nicotinamid, EX-4; Matrigel (14 to 28 days)	Nicotinamide + Matrigel (4–14 days)		
[86]	Activin A (EB) (6 days)	RA (EB) (1 day)	FGF7 (DMEM/B27) (3 days)	FGF7 + GLP-1+ Nicotinamide) (DMEM/B27) (4 days)	–		
[87]	Activin A, WNT3A, BMP4, VEGF, FGF-2) (RPMI) (2 days)	SFD + FGF10 + WNT3A ± DM (3 days)	Noggin + CYC + RA + FGF10 (DMEM) (3 days)	SB + Noggin (DMEM) (4 days)	SFD, SB, Noggin, Gamma SIX (9 days)		
[42]	Activin A	Activin A + FBS (RPMI) (3 days)	Dorsomorphin	Forskolin	–		
	CHIR99021)		Retinoic acid	Dexamethasone			
	(RPMI) (FBS)		SB431542 (7 days)	Alk5 inhibitor II			
	(1 day)		–	Nicotinamide (11 days)			
[88]	Activin A, Wnt 3A	Activin A (RPMI + FBS + ITS) (3 days)	KGF + TGF-β RI kinase Inhibitor IV	TT + CYC + Noggin (DMEM/B27)	Noggin + KGF + EGF		
	(RPMI + FBS + ITS)		(RPMI + FBS + ITS) (3 days)	(3 days)	(4+ days)		
[81]	GDF8	FGF7	FGF7, VitC, RA, SANT	FGF7, VitC, RA	Stage 5	Stage 6	Stage 7
	GSK3β inh	VitC	TPB, LDN (2 days)	SANT, TPB	SANT, RA	ALK5 inh II	ALK5 inh II
	(3 days)	(2 days)	–	LDN (3 days)	ALK5 inh II	T3, LDN	T3, *N*-Cys
	–	–	–	–	T3 LDN	GS inh XX	AXL inh
	–	–	–	–	(3 days)	(7–15 days)	(7–15 days)

### 5.1. Activin A

Activin A is a non-glycosylated cytokine that belongs to the TGF β family [89,90] and is actively involved in various biological processes, including wound repair, hemopoiesis, and differentiation [56,91]. Because of its unique properties, activin A plays important functional roles in diverse biological systems; These roles include differentiation into pancreatic [92], mesoderm [93], neural [94], erythroid [95], and pituitary cells [96]. Considerate and systemic effort has been applied to use activin A for pancreatic development, specifically entailing the formation of definitive endoderm [97,98]. Activin A is a homodimer of two β -A subunits that is normally not expressed at the gastrulation stage of the embryo. It does, however, signal through the same receptor complex as nodal, which is a BMP-type molecule that is expressed at high levels in the node. Several researchers tried to enhance insulin production using activin A on the basis of stem cell engineering. D’Amour *et al.* [32], Johannesson *et al.* [67], and Wang *et al.* [99], added activin A, along with WNT3A, to the culture medium of hESCs and found improved formation of the meso-endodermal state during the first stage of pancreatic differentiation (Table 3). Molecules that enhance insulin production are being pursued by continuous screening of molecules that may supplement activin A during endoderm induction. Cai *et al.*, reported that activin A induction of hESCs, along with the addition of retinoic acid, prompted expression of the gene *PDX1* in over 70% of cells in culture [65]. Therefore, activin A has generated unforeseen interest in the development of protocols focused on endoderm induction. Jiang *et al.*, have shown that activin A, with sodium butyrate, induces differentiation of hESCs to pancreatic cells during the early stages of endoderm formation [71]. However, the exact mechanism by which sodium butyrate treatment in combination with activin A induces differentiation during endoderm development is unclear. It is important to note that fetal calf serum was not a prerequisite in the protocol when activin A and sodium butyrate were used.

In recent years, the focus of research has largely shifted to development of a highly efficient step-wise protocol to direct pancreatic differentiation from hESCs using a combination of activin A and wortmannin to induce definitive endoderm formation [70]. Sustained exposure to high levels of activin A induces endoderm formation [97,98,99,100]. Jiang *et al.*, performed pancreatic differentiation of hESCs with minor modification to a previous combination of activin A and wortmannin and found better induction during endoderm formation [101]. The versatility of activin A is demonstrated in its specification of the anterior primitive streak region of meso-endoderm at an initial stage when cells are cultured in the presence of WNT3A, FGF2, and activin A [31]. Additional studies have revealed that further treatment of embryoid bodies (EBs) formed from dissociated hESCs with activin A promotes the expression of *FOXA2* and *SOX17* mRNAs, which are markers of definitive endoderm (Table 1 and Table 3) [102]. Activin A is also known to be a potential factor for the differentiation of hESCs into definitive endoderm (Table 3) [98].

Van Hoof *et al.*, studied differentiation into definitive endoderm using a medium conditioned by activin A-secreting CHO cells, rather than a medium supplemented with purified activin A [103]. Xu *et al.*, demonstrated pancreatic differentiation with three molecules, *i.e.*, activin A, FGF-2, and BMP4, in a serum-free medium without insulin [85]. Furthermore, Xu *et al.*, demonstrated that activin A with insulin in the culture medium did not affect the induction of endoderm markers such as *GSC* and *MIXL1* (Table 1) [85]. However, expression of other endoderm markers such as *SOX17* and *FOXA2* was drastically decreased [85]. Future efforts are underway to differentiate hESCs towards definitive endoderm using three different activin A-based treatments [104]. Thus, it can be summarized that activin A plays an important role in endoderm formation and will be useful for stem cell engineering.

### 5.2. Fibroblast Growth Factor (FGF)

FGF regulates differentiation and migration, promoting proliferation during embryonic development [105]. The optimal secretion of FGF, combined with that of other small and large molecules, not only leads to differentiation but also increases the number of hESCs that differentiate into β-cells. Seven different receptors play important roles in the FGF signaling pathway mediated by the four main tyrosine kinase receptors FGFR1, FGFR2, FGFR3, and FGFR4 [106]. Therefore, FGF promotes a close developmental relationship between the pancreas and other organs such as the liver, thyroid, and lung. FGF signals from the cardiac mesoderm to the ventral bud promote liver growth, whereas a pancreatic cell fate is triggered in the absence of FGF-2 [107,108,109,110]. Furthermore, the effect of FGF alone is insufficient, but the addition of optimal FGF along with a liver inhibitor results in pancreatic differentiation of hESCs. However, although FGF plays a vital role in pancreas formation, the function of the FGF signaling pathway is not yet fully understood. Eighteen different FGFs influence expression of the various growth factors involved in the regulation of pancreatic cell expansion. Lack of FGFs in culture medium strongly affects differentiation into pancreatic tissue from ES cells [107,108,111,112]. During complete formation of the pancreas, FGF binds to and activates various receptors, such as FGFRs (cytoplasmic tyrosine kinase enzymatic activity). The heparan sulfate proteoglycans (HSPG), a cysteine-rich FGF receptor (CFR), and FGFs activate ERK phosphorylation controlled by FGFR3 in pancreatic cell lines [113]. Expression of the *PDX1* transcription factor decreases when FGFR and MAPK signaling pathways are inhibited [114]. Several isoforms of FGFR, e.g., FGFR1b, FGFR1c, FGFR2b, FGFR2c, FGFR3b, and FGFR4, are expressed during pancreatic development. FGFR1 and FGFR4 are expressed early in pancreatic development, but their expression diminishes during adulthood [109].

FGF2 regulates specification of hESC-derived DE into different foregut lineages in a concentration-dependent and temporal manner. The specification of midgut endoderm into small intestine is completed during organ differentiation at high FGF2 levels. At low FGF2 concentrations, liver formation is promoted, whereas at higher concentrations, FGF2 represses *PDX1* expression and promotes lung formation. Pancreatic differentiation is promoted only by the addition of optimal levels of FGF. In the absence of FGFR signaling in hESCs, expression of *PDX1* is drastically affected [115,116,117,118]. Intermediate levels of FGF boost the expression of transcription factor such as *PDX1* and *NKX6.1* [114,119]. FGF4 expression in the posterior endoderm of the gastrula results in formation of gut endoderm at early embryonic stages. FGF4 promotes posterior endoderm formation by signaling through FGFR1c, FGFR2c, FGFR3c, and FGFR4 [120]. Although they are produced by mesoderm, FGF1 and FGF2 are also involved in gut endoderm formation. Miralles *et al.* [121], revealed that FGFR2 IIIb and its ligands FGF1, FGF7 [85], and FGF10 [122] are strongly expressed throughout pancreatic development.

The biological and molecular crosstalk among FGFs and retinoic acid indicates not only that retinoic acid plays an essential role in dorsal pancreas specification, but also that the addition of retinoic acid to the culture medium induces expression of FGF8, FGFR1, and FGFR4 in hESC-derived cells [67]. Although retinoic acid function is known to be crucial during the formation of the pancreas, the optimization of retinoic acid and FGF4 treatment resulted in 32% of all cells expressing the *PDX1* transcription factor, which activates the outgrowth of foregut endoderm during pancreatic differentiation [103]. FGF10 has a significant effect on the differentiation of MSCs into pancreatic epithelium [107,112,123]. Several factors are involved throughout dorsal pancreas development, among which early factors such as activin A and FGF are produced by the notochord [124]. Signals of FGFs from the cardiac mesoderm to the ventral bud promote liver growth, whereas a pancreatic cell fate is triggered in the absence of FGF-2. Therefore, the addition of optimal FGFs along with an inhibitor of liver fat helps to induce pancreatic differentiation from hESCs. D’Amour *et al.*, observed that FGF10 is essential during pancreatic induction, along with the hedgehog-signaling inhibitor KAAD-cyclopamine [32]; These molecules produced a 160-fold increase in the expression level of insulin mRNA during the differentiation of pancreatic cells. These cells rapidly express high levels of *HNF6*, *HLXB9*, and *PDX1* in the final stage of pancreatic differentiation [123,125,126,127]. Jiang *et al*., promoted pancreatic differentiation by the addition of FGF-2 along with noggin to terminate the induction of liver formation [30]. Johannesson *et al.*, observed that FGF is unable to induce *PDX1* expression with low *INS* expression in the absence of retinoic acid [67]. Cai *et al.*, followed a simple protocol with FGF7 to try to optimize the differentiation of hESCs and achieved more than 70% expression of *PDX1* gene in hESC-derived cells [65].

Several studies that have induced hESCs to differentiate into pancreatic cells utilized the interplay of several factors from multiple signaling pathways. Deutsch *et al.* [128], and Zaret and Grompe [129], observed that high BMP concentration in the culture medium is necessary during the formation of the liver, whereas low BMP concentration and FGF are necessary for the differentiation of pancreatic cells from hESCs. However, the FGF concentrations present during the formation of the foregut may not be appropriate because FGF expressed in the mesoderm in the budding stages is involved in the specification of several endodermal derivatives, such as the lung, pancreas, and stomach [130]. Recently, several researchers such as D’Amour *et al.* [32], Jiang *et al.* [71], Kroon *et al.* [39], Cai *et al.* [65], Johannesson *et al.* [67], Vallier *et al.* [55], Zhang *et al.* [70], and Mfopou *et al.* [68], followed a five-stage protocol (Figure 3) and added optimized levels of FGF along with other factors such as retinoic acid, noggin, SB431542, EGF, KAAD-cyclopamine, EX4, and Compound E after the formation of definitive endoderm (Table 3). These researchers studied and improved the production of β-cells during pancreatic differentiation at various expression levels of different transcription factors [32,39,55,65,67,68,70,71]. The reason for the importance of FGF is still unknown, although the presence of FGF was significant for *PDX1* expression. Further investigation of FGF during pancreatic differentiation will help to reveal the mechanism with greater precision.

### 5.3. Retinoic Acid

Retinoic acid is produced in the mesoderm during gastrulation by an enzyme called retinaldehyde dehydrogenase. In both mouse and human ES cell differentiation into pancreatic cells, retinoic acid is required for *PDX1* gene expression [32,39,55,65,67,68,70,71]. It aids in the formation of endoderm and regulates early stages of pancreatic differentiation from hESCs. The development of the pancreas and liver requires retinoic acid signaling via retinoic acid receptors. Retinoic acid induces the *PDX1* gene and regulates pancreatic development, but the implications of signal transduction by retinoic acid receptors have not been sufficiently studied despite the wide usage of retinoic acids. Without better knowledge of retinoic acid receptors, the function of this molecule will not be helpful in identifying new molecules involved in differentiation of ESCs towards pancreatic β-cells. Several researchers, such as D’Amour *et al.* [32], Jiang *et al.* [71], Kroon *et al.* [39], Cai *et al.* [65], Johannesson *et al.* [67], Vallier *et al.* [55], Zhang *et al.* [70], and Mfopou *et al.* [68], have worked on pancreatic differentiation using retinoic acids, but expression of the *PDX1* gene decreases when retinoic acid alone is used, whereas the combination of retinoic acid with other molecules increases *PDX1* gene expression significantly (Table 3). Shi *et al.*, observed that progressive treatment in a three-step approach with the combination of activin A, retinoic acid, and nicotinamide is required for pancreatic differentiation [79]. Mfopou *et al.*, studied the expression of the *PDX1* gene and found that it increased by up to 80% when noggin was supplemented with retinoic acid [68].

Noggin acts as a BMP and Smad1/5/8 inhibitor, and the optimal amount of retinoic acid drastically reduces the formation of liver cells [39,71,131]. Transcription factors such as NGN3, INS, and GCG fail to be expressed if retinoic acid is not added to the β-cell differentiation medium [32]. The efficacy of this combination was also shown in iPS cells by Zhang *et al.* [70]. In the combination of retinoic acid and activin A, suppression of the transcription factor *Shh* is important for the induction of pancreatic markers such as *amylase 2*, *insulin II*, *glucagon*, *PDX1*, and *Ppy*. Nakanishi *et al.*, showed that use of RA and activin A in floating culture could induce differentiation into insulin-producing cells [132]. Cai *et al.*, demonstrated that the presence of noggin and absence of retinoic acid in the culture medium of hESCs failed to promote production of *PDX1* [65]. The addition of FGF and noggin along with retinoic acid enhances *PDX1* gene expression effectively.

### 5.4. KAAD-Cyclopamine (CYC)

Pancreatic lineage specification consists of several stages and is associated with a cocktail of several small and large molecules, including cyclopamine. Systematic administration of cyclopamine inhibits the sonic hedgehog pathway [73,133,134,135]. Plant-derived cyclopamine inhibits the membrane protein smoothened to block the hedgehog signaling pathway [136,137,138,139]. The inhibition of liver formation leads to pancreatic development. The addition of FGF10 along with cyclopamine, retinoic acid, and indolactam V (ILV) promotes primitive gut formation and also results in high expression of certain markers, such as *PDX1*, *NEUROD1*, and *NGN3*. Similarly, Green *et al.*, developed β-cells from both hESCs and hiPSCs, by using cyclopamine as a hedgehog inhibitor [140]. Several studies have shown that cyclopamine plays a major role in reducing the tumor burden in pancreatic cancer by influencing the sonic hedgehog pathway [133]. Jaramillo *et al.*, demonstrated that pancreatic specification is achieved by inhibition of sonic hedgehog signaling by the addition of cyclopamine, whereas expression of *PDX1* is dramatically high because of the presence of cyclopamine along with retinoic acid [135]. Many researchers have used cyclopamine in a pancreatogenic molecule cocktail to produce a stepwise protocol for the formation of insulin-producing cells [32,39,67,68,141,142]. However, increasing the concentration of cyclopamine leads to increases in Wnt and β-catenin and causes colon cancer [143].

### 5.5. Wortmannin

Wortmannin is a molecule similar to activin A that directly inhibits the PI3K pathway [144], thereby indirectly promoting pancreatic development of ESCs [52]. PI3K inhibitors such as wortmannin and LY294002 have been identified by Powis *et al.* [53], Mclean *et al.* [51], and Vlahos *et al.* [54], respectively, and added to the medium, thereby initiating the nodal and TGFβ signaling pathways during definitive endoderm formation. Similarly, activation of the PI3K signaling pathway by cadherins is also inhibited by wortmannin [52,70], LY294002, and an Akt inhibitor [51]. Jeon *et al.*, generated differentiated β-cells from hiPSCs [47]; Wortmannin was added along with activin A at the first stage of differentiation to cause the rapid expression of various marker genes, such as *SOX17*, *HNF-3*, *CXCR4*, *GATA4*, and *FOXA2.* The addition of wortmannin along with activin A enhances the secretion of insulin by both hESCs [70,101] and hiPSCs [70].

### 5.6. Sodium Butyrate

Sodium butyrate is a short-chain fatty acid that acts as a histone deacetylase inhibitor to inhibit the dedifferentiation process [145,146,147]. Sodium butyrate, along with activin A, promotes the early stages of pancreatic development during the differentiation of insulin-producing cells using hESCs [71]. The early effect of sodium butyrate on differentiation leads to secretion of huge quantities of both glucagon and insulin [148]. The combination of activin A and sodium butyrate resulted in to the development of DE from mesenchymal murine adipose tissue in studies of differentiation into β-cells [149]. Moreover, the removal of sodium butyrate from the medium decreases the expression of *PDX1* [150]. However, no effects were observed in the presence of sodium butyrate alone, whereas the combination of sodium butyrate and activin A resulted in the formation of definitive endoderm and higher expression of *FOXA2* and *HNF4α* [71]. DeAizpurua *et al.*, observed that *MLK-1* gene expression was increased in the presence of sodium butyrate [151]. Similarly, the addition of sodium butyrate induces hESCs to form early-stage DE during pancreatic development [104,152]. The production of insulin by insulin-secreting cells is increased during differentiation by the presence of sodium butyrate in combination with GLP1 [153,154]. The *PDX1* and *NGN3* genes are highly expressed when small amounts of sodium butyrate are added, whereas transthyretin and antitrypsin was distinctly expressed at higher concentrations of sodium butyrate, which indicates that sodium butyrate promotes either liver or pancreatic cell fate depending on the concentration and the length of exposure [155].

### 5.7. Betacellulin

Betacellulin, a member of the EGF family [156], enhances the production of insulin-secreting cells when combined with activin A. Several researchers have demonstrated that betacellulin acts as an important modulator of β-cell growth, has a mitogenic effect on INS-1 cells [157], and is a ligand for Epidermal growth factor receptor (EGFR) and *erb*B-4 [35]. The injection of betacellulin into streptozotocin-diabetic and alloxan-diabetic mice stimulates β-cell neogenesis [158], whereas *NGN3* and betacellulin reverse streptozotocin-induced diabetes *in vivo* [159]. Similarly, the combination of activin A and betacellulin converts amylase-secreting pancreatic cells into insulin-positive cells [160]. *PDX1* expression is sustained during β-cell differentiation of hESCs by the addition of betacellulin and nicotinamide, whereas either of them alone is not sufficient in this process [84]. Nicotinamide also promotes regeneration [161], proliferation, and differentiation of insulin-secreting cells [162].

### 5.8. Noggin

Inhibition of BMP by noggin [163] promotes pancreatic development at a later stage of differentiation (*i.e.*, from primitive gut tube formation to pancreatic endoderm), whereas overexpression of noggin leads to severe pancreatic hypoplasia [164]. The expression of noggin along with FGF and retinoic acid promotes the induction of *PDX1* and other transcription factors, such as *FOXA2*, *HNF6*, and *SOX9*, during differentiation of hESCs into β-cells [68]. The addition of noggin promotes the formation of the pancreas and suppresses liver formation [165]. When only noggin and ALK5i were added to the medium during the pancreatic endoderm stage, expression of *NKX6*.1 increased fourfold, whereas 50-fold up-regulation of *NKX6.1* was observed when noggin and ALK5i were added at the same stage in the presence of a PKC activator. Furthermore, the combination increased the expression of various transcription factors, such as *PDX1*, *NGN3*, *NEUROD1*, and *PTF1α* [166]. Supplementation of noggin at the progenitor stage induced higher expression of *HNF4α* and *PDX1*, and removal of noggin after the progenitor stage led to generation of more α-cells [31,167].

### 5.9. EGF, HGF, KGF, and IGF

EGF, HGF, KGF, and IGF are important factors used in β-cell differentiation medium. During differentiation from hESCs, EGF significantly promotes the expansion of pancreatic progenitors by augmenting the number of *PDX1*-positive cells threefold [70]. Differentiated β-cells increase their production of insulin when further treated with HGF [83,168]. Kroon *et al.*, added KGF after definitive endoderm formation of hESCs [39]. Several researchers are presently using these growth factors alone or in combination; for instance, HGF [65] or EGF [70,71] alone, EGF plus IGF [70,71], and IGF with HGF [32,68] have all been used. These growth factors stimulate or promote differentiation into β-cells during the formation of pancreatic progenitors.

## 6. Clinical Trials

Contemporary studies of differentiating hESCs, hiPSCs, and hMSCs answer the question of how to produce maximal insulin secretion based on the physiological conditions of diabetic patients [169]. Several advances, such as screening and selection of small and large molecules, genetic engineering, and nuclear reprogramming, have improved dramatically in the last two decades. However, immunological differences between the donor and host, tumor formation, and unsatisfactory response to glucose concentration are the major limitations during clinical trials. Wharton’s jelly-derived MSCs were differentiated and transplanted into diabetic mice, and subsequently it was found that glucose levels are normalized [170]. Recently, Kim *et al.*, compared the growth potential of four different types of MSCs and identified that periosteum-derived progenitor cells (PDPCs) were more promising than cells derived from adipose tissue, bone marrow, or Wharton’s jelly [171]. The selection of ESC-derived insulin-secreting cells cloned along with insertion of the Herpes thymidine kinase gene normalized the glucose levels and body weight of mice within six hours and four weeks, respectively [29]. Normal insulin was secreted when β-cells differentiated from human adipose tissue-derived MSCs were transferred into five patients, without any immune rejection [172]. However, failure of glucose response and production of insulin result from insufficient maturation during differentiation. Secreted insulin reduces blood glucose levels in the presence of GLP1 via a cAMP-dependent pathway [173]. Studies of cell–cell interactions between host cells and differentiated stem cells after implantation found that these interactions promote insulin secretion based on physiological processes [34]. The Novocell team protected human islets using a hollow fiber macro device, transplanted them into nine normal, type I, and type II diabetic recipients, and observed after two weeks that more than 90% of the islets were viable and protected from the human immune system [40]. A few clinical trials with hMSCs are ongoing in the USA and China. However, currently there is no clinical trial using hiPSCs [41,43,86,87,88,174]. Different types of challenges are faced for controlled studies when applying results from *in vitro* studies to *in vivo* studies, particularly for human clinical trials.

## 7. Conclusions

Stem cell therapy is promising for the treatment of diabetes [175,176,177,178,179]. However, there are still some major technical obstacles that need to be overcome such as immune rejection, determining when undifferentiated cells become differentiated cells exactly, and other genetic and molecular controls before pluripotent stem cell-derived cells can be used for human therapy. Understanding the signaling pathways and mechanisms of existing molecules will lead to more success in the differentiation of insulin-producing cells from hESCs and hiPSCs. There is a need to optimize the concentration of existing molecules and find new molecules to develop clear-cut, rapid protocols. Initially, after the transplantation of hESCs and hiPSCs, it is mandatory to know how closely cell–cell interactions can control insulin secretion, thereby preventing high or low production of insulin in patients. In addition, the study of genetic insertion and any new molecules involved in signaling from the host cells is necessary to maintain the shelf life of differentiated stem cells in humans. Studies must also evaluate whether the derived cells are capable of surviving and producing insulin when exposed to glucose for long periods of time. Finally, the immunological differences between the donor and recipient need to be removed, and advances in genetic engineering may aid in the prevention of tumor formations.
